# Supplementary material for: Increased visual and cognitive demands emphasize the importance of meeting visual needs at all distances while driving
Source: PLoS One. 2021 Mar 16;16(3):e0247254. doi: 10.1371/journal.pone.0247254 (PMC7963041; doi:10.1371/journal.pone.0247254)
Supplement: Attachment — Submitted filename: Response to reviewers.pdf [file pone.0247254.s001.pdf]

November 27rd, 2020

Rebutal letter

Dear reviewers,

Thank you very much for taking the time to read this manuscript entitled "*Increased visual and cognitive demands emphasize the importance of meeting visual needs at all distances while driving*" and leave us your feedback. You can read below, our comments under yours (in bold and italics). We have made some adjustments following your recommendations (cyan highlighting) as well as from our side (yellow highlighting). The underlined passages represent the passages addressed in the case of justification on our part.

Reviewer 1

1. In the experimental design, the 21 participants were selected with varying degrees of vision degradation, no normal vision participation, lack of control group. Our experimental methodology is based on a within participant design, each participant performing two blocks of two driving scenarios in optimal vision and degraded vision. Each block contains a rural scenario and a highway scenario with some slight modifications of the course of the events to avoid any learning. Our protocol was counterbalance (see lines 234-236). Half of the participants started the experimentation by degraded vision block, half of the participant by optimal vision. Between comparisons, have been done on the relative within effect between optimal and degraded vision. This way, each subject is his own control. This can also be seen in figure 5 (see line 245).

2. In the course of the experiment, whether each driver drives all the simulated scenes, continuous driving or random driving. Each driver performs two different, but equivalent rural scenario (average time duration of 12 min duration) and two times the same highway scenario (average time duration of 6 min 30 duration) with two different randomized set of 7 visual panels for the secondary task. Each scenario was performed in the two driving blocks: optimal vision and degraded vision. The order was counterbalanced for visual condition (optimal and degraded vision) and randomized for the order of the scenarios (rural first followed by the highway or vice versa in block 1 as well as in block 2) (see lines 234-237). For example, the participants could have the following driving sequence:

Typical example for one participant
Block 1. Optimal Vision: Rural scenario first and Highway scenario second
Block 2. Degraded Vision: Highway scenario first and Rural scenario second
Or
Typical example for a second participant
Block 1. Degraded Vision: Highway scenario first and Rural scenario second
Block 2. Optimal Vision: Highway scenario first and Rural scenario second

3. In the study, speed is the characterization parameter of cognitive mechanism, and it is suggested to explain its rationality and comprehensiveness. Speed is not the characterization parameter of cognitive mechanisms, but the characterization of the effect of our dependent variables. From the literature, it is well known that speed is a factor expressing reaction to cognitive load (Engström et al., 2017) or decreased visuo-cognitive capacities (Michaels et al. 2017).

Michaels and his colleagues (2017) observed a decreasing speed as strongly correlated with decreased visuo-cognitive capacities. Our methodology consists of asking the participant to drive at a constant velocity of 90 km/h, and increasing the cognitive and visual demands by performing a visual search task or by manipulating the mental workload as a function of the driving scenario rural or highway. Thanks to your comment, we have decided to clarify our hypotheses as well as to improve the introduction using justifications for the choice of our behavioral variables (see lines 132-136).

4. It is recommended to give a final conclusion on the effects of the interaction between degraded vision and cognitive mechanisms on driving behavior. These details are specified in the conclusion (see lines 712-713, 716-717,722-728). If the reviewer wishes, we are open to further details on the recommendation raised.

Reviewer 2

1.The authors need to clarify why only French speakers' volunteers or young participants were recruited. The specification that the participants are all French speakers is justified since the panels contain names of towns of French origin and etymology. This also helps to promote homogeneity in our sample and therefore allows a more adequate comparison. The rationale for favoring a sample of young drivers comes from the desire to avoid the cognitive degradation bias associated with aging. Indeed, this was notably observed in Michaels et al., 2017.

References

Engström J, Markkula G, Victor T, Merat N. Effects of cognitive load on driving performance: The cognitive control hypothesis. Human factors. 2017 Aug;59(5):734-64.

Michaels J, Chaumillon R, Nguyen-Tri D, Watanabe D, Hirsch P, Bellavance F, Giraudet G, Bernardin D, Faubert J. Driving simulator scenarios and measures to faithfully evaluate risky driving behavior: a comparative study of different driver age groups. PLoS one. 2017 Oct 10;12(10):e0185909.
