# Supplementary material for: Increased visual and cognitive demands emphasize the importance of meeting visual needs at all distances while driving
Source: PLoS One. 2021 Mar 16;16(3):e0247254. doi: 10.1371/journal.pone.0247254 (PMC7963041; doi:10.1371/journal.pone.0247254)
Supplement: Attachment — Submitted filename: Response to reviewer.pdf [file pone.0247254.s002.pdf]

January 19th, 2021

Rebuttal letter

Dear reviewer,

We would like to thank you for the thoroughness you have shown through the review of our manuscript entitled "*Increased visual and cognitive demands emphasize the importance of meeting visual needs at all distances while driving*". Your perspective allowed us to ensure that no relevant detail was left behind in the writing of our manuscript. We have made some adjustments following your recommendations (yellow highlighting in the revised manuscript document).

"In the course of the experiment, whether each driver drives all the simulated scenes, continuous driving or random driving."

Considering your perspective on the lack of explanations according to our protocol, we have decided to add some details to the protocol part, specifying our approaches to randomization and counterbalancing of the participant sequences as well as their interest, namely to avoid a learning effect due to the repetition of the scenarios between the driving blocks and to avoid a learning effect. Participants, in fact, performed two scenarios (rural and highway) followed by a break as well as an additional block of two scenarios. (see **lines 234-241**).

" It is recommended to give a final conclusion on the effects of the interaction between degraded vision and cognitive mechanisms on driving behavior, not just to analyze the data."

We have undertaken to reorganize the conclusion in order to put forward, as you have pointed out, the interpretive aspect of our results and not just their analysis (see **lines 720-747**).

We hope that with these latest changes our perspectives will come together so that we can publish an optimal version of our manuscript.

Regards,

Amigale Patoine, Laura Mikula, Sergio Mejía Romero, Jesse Michaels, Océane Keruzore, Romain Chaumillon, Delphine Bernardin and Jocelyn Faubert
